# Leigh Syndrome Due to *NDUFV1* Mutations Initially Presenting as LBSL

**DOI:** 10.3390/genes11111325

**Published:** 2020-11-09

**Authors:** Nurun Nahar Borna, Yoshihito Kishita, Norio Sakai, Yusuke Hamada, Koji Kamagata, Masakazu Kohda, Akira Ohtake, Kei Murayama, Yasushi Okazaki

**Affiliations:** 1 Diagnostics and Therapeutics of Intractable Diseases, Intractable Disease Research Center, Graduate School of Medicine, Juntendo University, Bunkyo-ku, Tokyo 113-8421, Japan; b-nurun@juntendo.ac.jp (N.N.B.); y-kishita@juntendo.ac.jp (Y.K.); m-kohda@juntendo.ac.jp (M.K.); 2 Child Healthcare and Genetic Science Laboratory, Division of Health Sciences, Graduate School of Medicine, Osaka University, Osaka 565-0871, Japan; norio@sahs.med.osaka-u.ac.jp; 3 Department of Pediatrics, Toyonaka Municipal Hospital, Toyonaka, Osaka 560-8565, Japan; yh5195@gmail.com; 4 Department of Radiology, Graduate School of Medicine, Juntendo University, Bunkyo-ku, Tokyo 113-8421, Japan; kkamagat@juntendo.ac.jp; 5 Department of Pediatrics & Clinical Genomics, Faculty of Medicine, Saitama Medical University, Moroyama, Saitama 350-0495, Japan; akira_oh@saitama-med.ac.jp; 6 Center for Intractable Diseases, Saitama Medical University Hospital, Moroyama, Saitama 350-0495, Japan; 7 Department of Metabolism, Chiba Children’s Hospital, Midori-ku, Chiba 266-0007, Japan; kmuraya@mri.biglobe.ne.jp; 8 Laboratory for Comprehensive Genomic Analysis, RIKEN Center for Integrative Medical Sciences, Yokohama, Kanagawa 230-0045, Japan

**Keywords:** leukodystrophy, leukoencephalopathy with brainstem and spinal cord involvement and lactate elevation, Leigh syndrome, *NDUFV1*, OxPhos deficiency

## Abstract

Leigh syndrome (LS) is most frequently characterized by the presence of focal, bilateral, and symmetric brain lesions Leukoencephalopathy with brainstem and spinal cord involvement and lactate elevation (LBSL) is a rare condition, characterized by progressive pyramidal, cerebellar, and dorsal column dysfunction. We describe a case with infantile-onset neurodegeneration, psychomotor retardation, irritability, hypotonia, and nystagmus. Brain MRI demonstrated signal abnormalities in the deep cerebral white matter, corticospinal and dorsal column tracts, and pyramids, which resemble the MRI pattern of a severe form of LBSL, and involvement of basal ganglia and thalamus that resemble the radiological features of LS. We identified biallelic loss-of-function mutations, one novel (c.756delC, p.Thr253Glnfs*44) and another reported (c.1156C > T, p.Arg386Cys), in *NDUFV1* (NADH:Ubiquinone Oxidoreductase Core Subunit V1) by exome sequencing. Biochemical and functional analyses revealed lactic acidosis, complex I (CI) assembly and enzyme deficiency, and a loss of NDUFV1 protein. Complementation assays restored the NDUFV1 protein, CI assembly, and CI enzyme levels. The clinical and radiological features of this case are compatible with the phenotype of LS and LBSL associated with *NDUFV1* mutations.

## 1. Introduction

Complex I (CI) is a mitochondrial oxidative phosphorylation (OxPhos) subunit, and its dysfunction causes a defect in energy production that affects various organs. NADH:Ubiquinone Oxidoreductase Core Subunit V1 (NDUFV1) (also called NADH Dehydrogenase (Ubiquinone) Flavoprotein 1) is a nuclear-encoded structural subunit of CI [1]. Clinically, children with *NDUFV1* mutations show developmental regression, epilepsy, hypotonia, spasticity, dystonia, ophthalmoplegia, strabismus, and nystagmus [2,3,4]. The association of *NDUFV1* mutations with Leigh syndrome (LS), Leigh-like syndrome (LL), and diffuse leukoencephalopathy with or without cavitation have been reported [4,5]. There are cases reported to be associated with leukodystrophy and myoclonic epilepsy, and episodic leukoencephalopathy due to *NDUFV1* mutations [6,7].

Leukoencephalopathy with brainstem and spinal cord involvement and lactate elevation (LBSL, MIM#611105) is a novel form of leukoencephalopathy, characterized by cerebral and cerebellar white matter abnormalities, and dysfunction of pyramidal tracts, lateral corticospinal tracts, and the dorsal column [8]. Distinct radiological findings include selective involvement of the brainstem and spinal cord tracts. Magnetic resonance spectroscopy (MRS) detection of lactate elevation in the abnormal white matter is also characteristic, but it can be normal in some cases [9]. The clinical spectrum of LBSL can vary, from early-onset severe disease to an adult-onset disease with a slow course. Variable clinical manifestations, including delayed motor development, spasticity, ataxia, cognitive impairment, epilepsy, dysarthria, learning difficulties, and peripheral neuropathy may occur [8]. The phenotypic variations of the disease are much more diverse than initially described, and the disease course is slow with low mortality, except in infantile-onset cases [10]. To date, LBSL has been reported to be associated with mutations in the mitochondrial aspartyl-tRNA synthetase 2 gene (*DARS2*) [10], and the iron-sulfur cluster assembly 2 (*ISCA2*) gene [11].

The present case highlights a subject with biallelic *NDUFV1* mutations with leukodystrophy and clinical and radiological evidence of LS and LBSL. This is the case of an infantile-onset LS/LBSL associated with *NDUFV1* mutations, which provides further information on the phenotypic variability of mitochondrial diseases caused by *NDUFV1* mutations. Our findings underscore the importance of a multigene panel for the molecular diagnosis of suspected mitochondrial diseases. 

## 2. Materials and Methods

### 2.1. Study Approval

The study was approved by the ethics committee of Saitama Medical University and was performed after receiving written informed consent from the parents of the subject.

### 2.2. Neuroimaging

Brain MRI and CT scans of the subject were performed several times. Here we describe neuroimages at the age of 3 and 13 years.

### 2.3. Genetic Analysis and Sanger Sequence Validation

Exome analysis was performed as previously described [12]. Whole-exome sequencing was performed using the HiSeq2500 (Illumina, San Diego, CA, USA) platform, and for the alignment of the sequencing reads the NCBI human genome reference (GRCh37/hg19) was used. Sanger sequencing was performed on genomic DNA prepared from the subject’s fibroblasts and the blood of the parents for validation of prioritized variants. The ABI3130XL and BigDye v3.1 Terminator (Applied Biosystems, Foster City, CA, USA) were used for sanger validation. Sequencing primers were: Chr11_67378517-F: 5′–GCAATGGGCATCTCTGGAGT–3′ and Chr11_67378517-R: 5′–TCTCTTTGTGGACACCTGCC–3′; Chr11_67379443-F: 5′–GCTGGAGGAGGCCAGAAC–3′ and Chr11_67379443-R: 5′–GAAACGTGCCATCACCTTG–3′.

### 2.4. Cell Culture

We cultured fetal human dermal fibroblasts (control 1; Toyobo, Osaka, Japan), neonatal human dermal fibroblasts (control 2; Toyobo), and the subject’s fibroblasts at 37 °C and 5% CO_2_ in Dulbecco’s modified Eagle’s medium (DMEM; Nacalai Tesque Inc., Kyoto, Japan) supplemented with 10% fetal bovine serum (Sigma-Aldrich, St. Louis, MO, USA) and 1% penicillin-streptomycin (Nacalai Tesque Inc.). 

### 2.5. SDS-PAGE, BN-PAGE, and Immunoblotting

Mitochondrial (MT) extracts were prepared from fibroblast pellets. Cells were suspended in MT isolation buffer A (220 mM mannitol, 20 mM HEPES, 70 mM sucrose, 1 mM EDTA, pH 7.4, 2 mg/mL bovine serum albumin, 1 × protease inhibitor cocktail) and homogenized by a Dounce homogenizer on ice. Homogenates were centrifuged at 700 *g* for 5 min, and at 10,000 *g* for 10 min at 4 °C. Resultant MT pellets were washed twice with MT isolation buffer B (220 mM mannitol, 20 mM HEPES, 70 mM sucrose, 1 mM EDTA, pH 7.4, 1 × protease inhibitor cocktail) and used for the downstream experiments. Total cell lysates (TCLs) were prepared using M-PER™ Mammalian Protein Extraction Reagent (Thermo Fisher Scientific, MA, USA). Twenty micrograms of mitochondrial or TCL proteins were separated by electrophoresis on 10% SDS-PAGE gel. 

Blue native (BN)-PAGE was performed to separate individual OxPhos complexes. MT fractions were solubilized in 1% Triton X-100 and separated using the 4–16% NativePAGE Novex Bis-Tris Gel System (Life Technologies, Warrington, UK). The anti-NDUFV1 antibody was from Sigma (AV48312). The primary antibodies used in this study were: NDUFA9 (Life Technologies, 459100), SDHA (Life Technologies, 459200), UQCRC1 (Life Technologies, 459140), COXI (Life Technologies, 459600), HSP60 (Abcam, Cambridge, UK, ab46798), and ACTB (β-actin; Genetex, Irvine, CA, USA, GTX124388). The secondary antibodies used were: Anti-Mouse (GE Healthcare, Chicago, IL, USA, NA931) and Anti-Rabbit (Promega, Madison, WI, USA, P044901) IgG.

### 2.6. OxPhos Enzyme Activity Assays

Spectrophotometric enzyme activity assays using MT from fibroblasts and lentiviral-mediated transduced samples were performed as previously followed [12].

### 2.7. Lentivirus-Mediated Complementation Assay

Total RNA isolation from fibroblasts, cDNA synthesis, and construction of plasmids were performed as described previously [12]. PCR was performed to amplify *NDUFV1* (NM_007103.4) from control and subject’s cDNA. The primer sequences used to clone *NDUFV1* were: *NDUFV1*-F: 5′–AGTGGCGGCCGctcgagCCACCATGCTGGCAACACGGCGG–3′ and *NDUFV1*-R: 5′–TAGGCTTACCctcgagCTAAGAGGCAGCCTGCCGGGCCTG–3′.

### 2.8. Statistical Analysis

Statistical analysis was performed using two-tailed Student’s *t*-test, ** *p* < 0.01 and *** *p* < 0.001 were considered statistically significant.

## 3. Results

Case description:

The female subject was born to non-consanguineous Japanese parents after full-term pregnancy, without remarkable complications. At birth, her weight was 2458 g and she had normal developmental milestones until seven months. She then presented with feeding difficulty, excessive crying, and irritability. On examination, hypotonia, spasticity, myoclonus, nystagmus, and reduced deep tendon reflexes were observed. She was unable to roll over, hold her head, or sit up by herself. At nine months old, she developed slurring of speech, hyperacusis, hepatomegaly, and gradual loss of subcutaneous fat. MRI of the brain revealed periventricular white matter lesions, and Krabbe disease was considered as a possible diagnosis. Genetic analysis did not identify any mutation in Krabbe-related genes. Laboratory findings revealed elevated levels of blood lactate (42 mg/dL) (normal range: 4.8–19.8 mg/dL) and pyruvate (2.6 mg/dL) (normal range: 0.7–1.4 mg/dL). On suspicion of mitochondrial dysfunction, dichloroacetic acid and vitamin B1 were prescribed.

**Figure 1:**
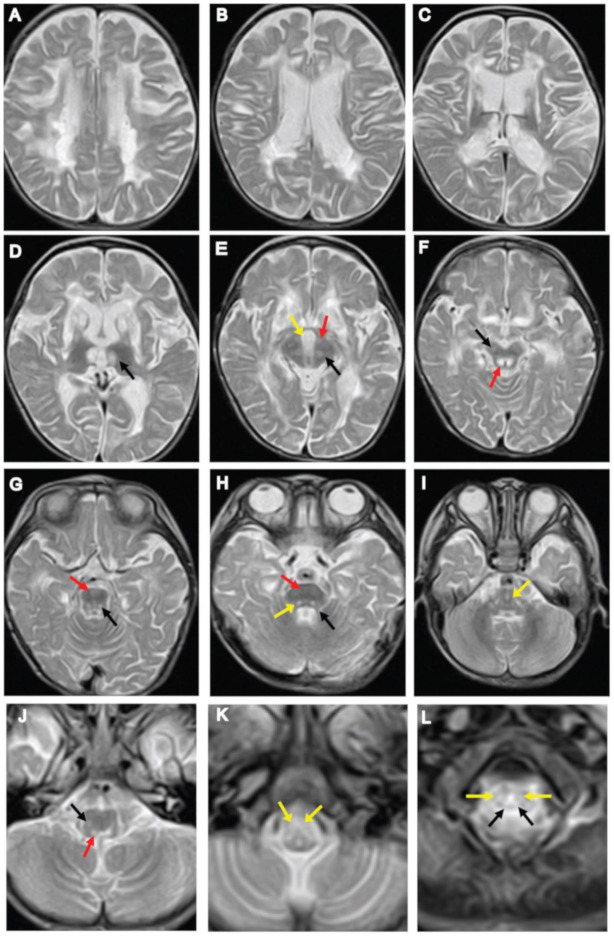
Neuroimaging findings at three years of age. (**A**–**C**) Axial T2-weighted images of brain MRI showing signal abnormalities in the periventricular white matter, putamen, caudate nucleus, splenium of the corpus callosum, and anterior and posterior limb of the internal capsule. (**D**) Symmetrical bilateral areas of increased signal intensity in the medial thalamic nucleus (black arrow). (**E**,**F**) Axial T2-weighted images of the midbrain: (**E**) interpeduncular cistern (yellow arrow), cerebral peduncle (red arrow), substantia nigra (black arrow); (**F**) substantia nigra (black arrow) and periaqueductal region (red arrow). (**G**–**I**) T2-weighted images of the pons: (**G**) pyramidal tract (red arrow), pontine tegmentum (black arrow); (**H**) superior cerebellar peduncle (black arrow), pyramidal tract (red arrow), pontine tegmentum (yellow arrow); (**I**) diffuse hyper-intensive lesions in the medial lemniscus (yellow arrow). (**J**,**K**) T2-weighted images of the medulla oblongata shows abnormal intensities in the medullary tegmentum, spinocerebellar tracts, and pyramids: (**J**) medullary tegmentum (red arrow), spinocerebellar tract (black arrow); (**K**) pyramids (yellow arrow). (**L**) T2-weighted images of the upper cervical spinal cord revealed abnormal intensity within the lateral corticospinal tracts (yellow arrows) and the dorsal columns (black arrows).

**Figure 2:**
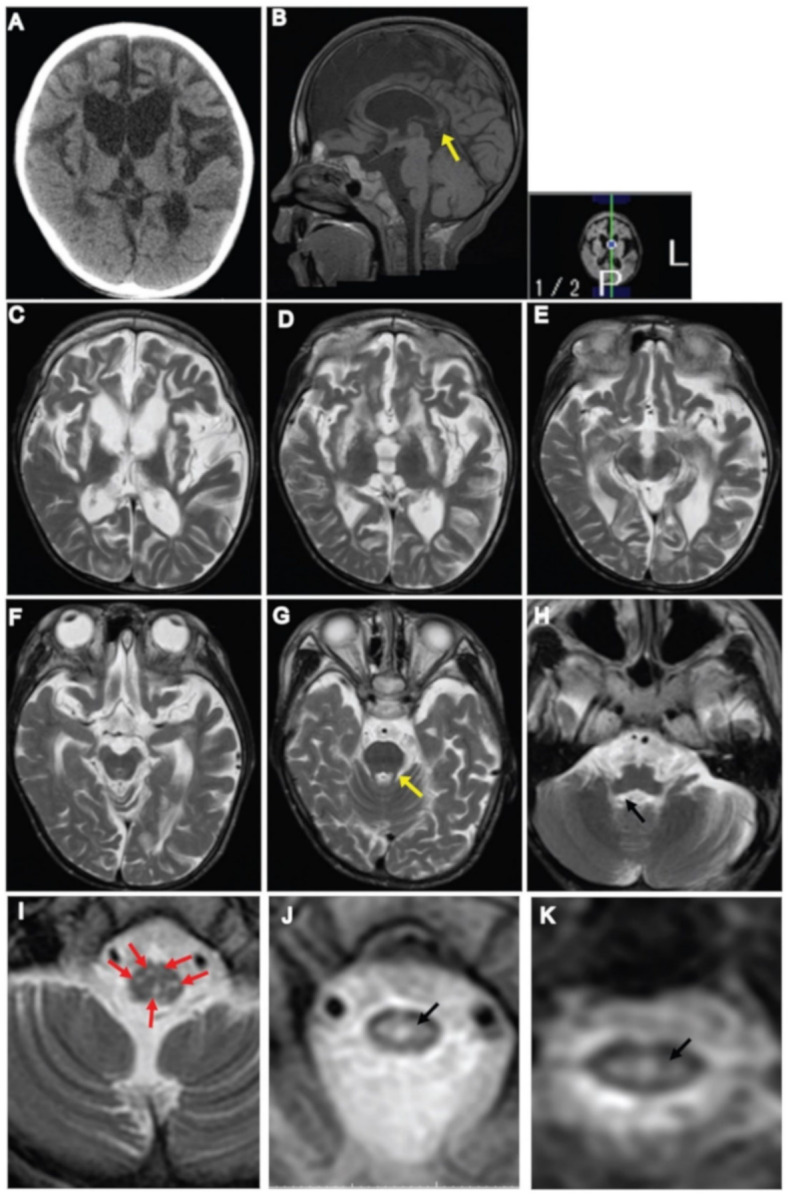
Neuroimaging findings at 13 years of age. (**A**) CT scan of the brain showing cerebral atrophy and ventricular enlargement. (**B**) Sagittal T1-weighted image of the brain showing ventricular enlargement, thinning of the corpus callosum with foci in the posterior body of the corpus callosum (arrow). (**C**–**E**) Axial T2-weighted images of the brain showing signal changes in the periventricular and frontal white matter, the putamen, caudate nucleus, corpus callosum, and anterior limb of the internal capsule. (**F**–**I**) Within the brainstem: (**F**) no signal abnormality was observed in the midbrain; (**G**) superior cerebellar peduncle (yellow arrow); (**H**) inferior cerebellar peduncle (black arrow); (**I**) T2-axial images of the medulla showing spotty high signal in the medulla (arrows), however pyramids and medial lemniscus decussation were preserved. (**J**,**K**) T2-axial image of upper cervical spinal cord showing high intensity area in the central part of the spinal cord (arrow).

At three years of age, brain MRI showed irregular signal abnormalities in the putamen, caudate nucleus, corpus callosum, anterior and posterior limb of the internal capsule, and the thalamus (Figure 1A–D). In the brainstem, signal abnormalities in the pyramidal tract and medial lemniscus were observed throughout the pons and medulla (Figure 1G–K). Hyperintensities in the interpeduncular cistern, substantia nigra and periaqueductal region were also observed in the midbrain (Figure 1E,F). Signal abnormalities in the superior cerebellar peduncle, spinocerebellar tract, medullary and pontine tegmentum were observed (Figure 1G–J). White lesions in the lateral corticospinal tract and dorsal column tracts of the spinal cord were observed (Figure 1L). Subsequent MRI of the brain at 13 years of age, showed irregular, spotty signal abnormalities in the putamen, caudate nucleus, and corpus callosum, which extended into the anterior limb of the internal capsule (Figure 2C–E). Signal abnormalities in the midbrain were apparently reduced, and in the brainstem, superior and inferior cerebellar peduncle showed signal abnormalities (Figure 2F–H). T2-Axial images of the medulla showing spotty high signal in the medulla (Figure 2I), and upper cervical spinal cord (Figure 2J,K). Brain CT scan showed cerebral atrophy accompanied by ventricular enlargement (Figure 2A). Sagittal T1-weighted brain MRI showed enlargement of the ventricle and diffuse thinning of the corpus callosum (Figure 2B). MRS showed lactate levels in the cerebral white matter to be within the normal range.

**Figure 3:**
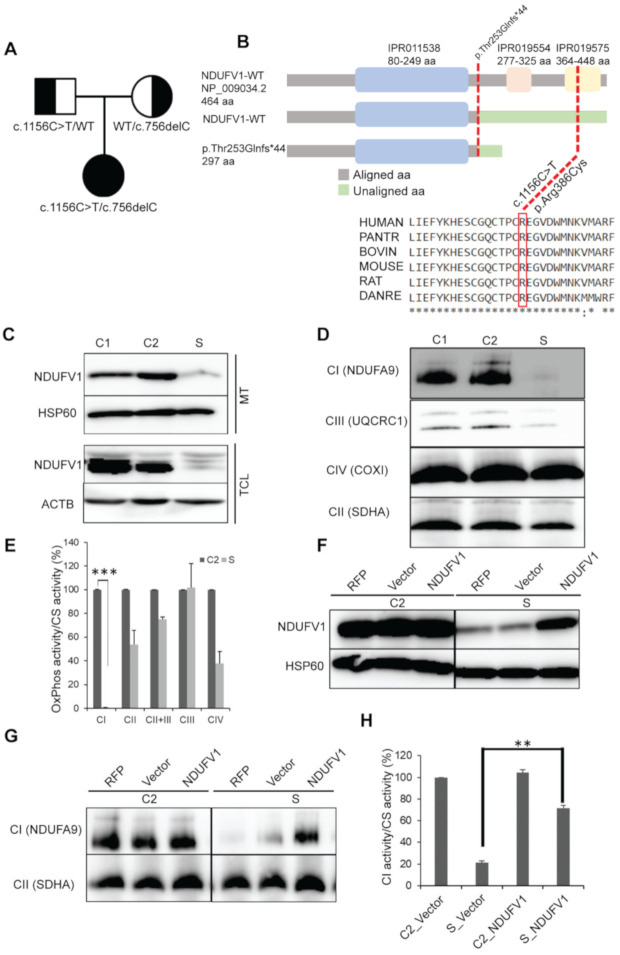
*NDUFV1* mutations detected in a subject. (**A**) Segregation of the family showing the inheritance pattern of the variants. (**B**) A graphical representation of NDUFV1 with domains illustrating the position of mutations (red dotted lines) (not to scale). Mutalyzer 2.0.32 (https://mutalyzer.nl/name-checker) for truncated protein prediction, InterPro database (https://www.ebi.ac.uk/interpro/protein) for protein domain prediction, and ClustalW (http://www.ebi.ac.uk/clustalw2) for amino acid (aa) alignment were used. Asterisks (*) indicates conserved aa; IPR011538, NADH: ubiquinone oxidoreductase 51kDa subunit, FMN-binding domain; IPR019554, Soluble ligand binding domain; IPR019575, NADH: ubiquinone oxidoreductase 51kDa subunit, iron-sulfur binding domain. (**C**) SDS-PAGE immunoblotting of mitochondrial (MT) and total cell lysate (TCL) showing loss of NDUFV1 protein in the subject (S) compared with controls (C1, fHDF; C2, NHDF). HSP60 and ACTB were used as loading controls. (**D**) BN-PAGE immunoblotting showing destabilization of CI assembly in S. Complex II was used as loading control. (**E**) OxPhos enzymology revealed CI enzyme deficiency in S compared to C2. Values are expressed as the mean ± SEM. Three independent experiments, two tailed student’s *t* test done and *** *p* <0.001 considered statistically significant. (**F**–**H**) Lentivirus-mediated complementation assays showing: F, restoration of the NDUFV1 protein in S; G, stabilization of CI assembly; H, restoration of CI enzyme activity. Values are expressed as the mean ± SEM. Three independent experiments, ** *p* <0.01 considered statistically significant RFP, mtTurbo-RFP; Vector, pCS-CA-MCS.

Identification of *NDUFV1* variants:

We identified a novel variant that causes protein-truncation (c.756delC, p.Thr253Glnfs*44) and a reported variant (c.1156C > T, p.Arg386Cys) in *NDUFV1* by whole-exome sequencing. Sanger sequencing was performed for validation, which confirmed the inheritance of the variants from the parents (Figure 3A). Protein domain prediction showed that p.Thr253Glnfs*44 resides outside of recognized domains and that p.Arg386Cys is in the iron-sulfur binding domain. Amino acid alignment showed high conservation of Arg386 among different species (Figure 3B).

Functional analyses:

To examine whether the variants are pathogenic, we analyzed NDUFV1 protein levels by Western blotting. Different fractions from the subject’s fibroblasts showed a significant reduction in NDUFV1 protein levels compared with that in controls (Figure 3C). BN-PAGE immunoblotting of Triton X-100 solubilized mitochondria revealed reduced expression of CI (NDUFA9) assembly (Figure 3D) and a significant reduction of CI enzyme activity in the subject (Figure 3E). We performed lentivirus-mediated complementation assays which showed that NDUFV1 protein levels were restored after transfection of the subject’s fibroblasts with wild-type *NDUFV1* cDNA (NM_007103). Transfection of the wild-type *NDUFV1* also stabilized CI assembly and restored CI activity (Figure 3F–H).

## 4. Discussion

Based on clinical and neuroimaging features, LBSL is a newly described leukoencephalopathy associated with a mitochondrial disorder [8]. In this study, we present a subject with *NDUFV1* mutations that initially presented clinical features and MRI abnormalities that resemble the syndrome of LBSL and LS, and later LS radiological features. This study highlights the phenotypic variability of mitochondrial disorders caused by *NDUFV1* mutations and underscores the importance of molecular diagnosis for mitochondrial diseases.

We present the MRI findings in our case and compare similarities and dissimilarities with LBSL and LS radiological diagnostic criteria in Table 1. The radiological findings in the present case are comparable with the major and minor brain MRI diagnostic criteria described previously [13]. Among the major criteria, inhomogeneous signal abnormalities in the cerebral white matter, pyramids and medial lemniscus at the level of the medulla oblongata, and dorsal columns and lateral corticospinal tracts of the spinal cord were observed (Figure 1). Among the minor criteria, signal abnormalities in the corpus callosum, internal capsule, superior cerebellar peduncles, and in the spinocerebellar tracts were observed (Figure 1). In this case, the thalamus was involved in early MRI images, but later hyperintensities were dispersed. The involvement of the globus pallidus, thalamus, and dentate nucleus is not included in LBSL diagnostic criteria; affected globus pallidus was observed in four patients with *DARS2* mutations and thalamus in a patient with the MRI criteria of LBSL with an unknown genetic cause [13]. Leigh syndrome typically shows bilateral symmetrical lesions within the brainstem and basal ganglia, periaqueductal region, and cerebral peduncles [14,15]. Brain lesions involving the white matter and cerebral atrophy have been reported [16]. In our case, MRI findings at three years of age show hyperintensity in the basal ganglia, thalamus, substantia nigra, cerebral peduncle, and periaqueductal region in the midbrain (Figure 1). These MRI findings are suggestive for LS. Findings in the brainstem have specific structure involvement, which is considered as more distinctive features of LBSL rather than LS (Figure 1). Therefore, we conclude that our patient shows the radiological diagnostic criteria of LS and LBSL at three years of age.

MRI findings at 13 years of age show regression of abnormalities observed in the previous MRI (Figure 2). Cerebral leukodystrophy, diffuse atrophy of the corpus callosum, lesions in the anterior limb of the internal capsule, basal ganglia, superior and inferior cerebellar peduncles, hyperintensity at the level of medulla, and central part of the spinal cord were involved. CT scan reveals severe cerebral atrophy, diffuse atrophy of the corpus callosum with foci in the posterior part, which was described previously in LBSL patients [18]. Although the regression in MRI abnormalities was observed at 13 years, a few LBSL resemble radiological features might be present that we cannot observe due to severe cerebral atrophy. Sharing a few features of two separate mitochondrial diseases are rare but exist. There is a report on an overlapping Leigh syndrome/myoclonic epilepsy with RRF in an adolescent patient due to m.8344A > G mutation [19].

The correlations between the disease severity and degree of gene dysfunction, or genotype-phenotype have not been established for LBSL. One study of *DARS2* missense mutations in LBSL patients has been shown that the mutations have variable effects on the enzyme activity, protein expression, localization, and dimerization of protein [20]. Another report observed that the same compound heterozygous mutations presented with phenotypic variability, from a healthy individual to disabled patients, within a family with *DARS2*-related LBSL [21]. Three siblings with LBSL who presented with nystagmus, slurring of speech, muscle tonus abnormality, ataxic gait, hypo or hyperreflexia, tremor, and mental retardation caused by *DARS2* mutation were considered to display a severe form of LBSL [22]. These siblings had extensive MRI findings for LBSL, with diffuse cerebral and cerebellar white matter abnormalities, and globus pallidus involvement. Furthermore, typical LBSL diagnostic features, like signal abnormalities in the posterior limb of the internal capsule, pyramidal tracts, medial lemniscus, cerebellar peduncles, dorsal columns, and lateral corticospinal tracts were also observed [22]. A comprehensive literature review indicates hypotonia, motor regression, feeding difficulty, and nystagmus in LBSL patients caused by *ISCA2* mutations [11]. Our subject also had neurophysiological findings, similar to other reported LBSL cases, such as irritability, hypotonia, hyporeflexia, nystagmus, slurring of speech, and feeding difficulty. Our case presented most of the typical features of infantile-onset severe form of LBSL, both clinically and radiologically.

The known *NDUFV1* c.1156C > T mutation is mostly associated with leukodystrophy [23], or LS with brainstem involvement [3]. A patient with biallelic mutations of *NDUFV1* (c.1156C > T, c.753delCCCC) showed an LS phenotype [4]. Patients with mitochondrial DNA mutations (m.3197T > C and m.3275C > G) presented with LS and spinal cord involvement have been reported [24,25]. There is also a report of a patient presented with LS and spinal cord involvement caused by *NDUFA1* mutations, and the brain MRI uncovered typical LS findings with lesions in the anterior column of the grey matter of the spinal cord [26]. There is no published report describing MRI features of LS/LBSL associated with *NDUFV1*. In our case, it is likely that the loss-of-protein-function is caused by the biallelic mutations; the frameshift mutation was predicted to be likely deleterious to protein function and contributed to the disease phenotype.

There is a possibility that rare diseases, such as LBSL, are under- or mis-diagnosed and there are no standard treatment guidelines for LBSL; only supportive treatments are offered to the affected individuals. Therefore, it is crucial to understand the pathophysiology of the genes and allelic variants involved, and the natural history of the disease process to determine drug regimens.

We confirmed that *NDUFV1* variants are pathogenic and the patient appeared to have mimicking features of both LBSL and LS. We hypothesize that the course of the phenotype of this case begins with some features of LS/LBSL and is followed by LS, which emphasizes that the same genetic abnormality can be associated with a wide spectrum of phenotypes.

## Figures and Tables

**Table 1:** Similarities and dissimilarities of MRI signals of the subject with LBSL and LS.

Neuroradiological Involvement (MRI Criteria)	LBSL (MRI Criteria)	LS (MRI Criteria)	MRI of the Subject (3 Years)	MRI of the Subject (13 Years)
Cerebral white matter	Affected (relative sparing of the subcortical white matter)	Unaffected/affected †	Affected	Affected
Corpus callosum	Genu, splenium, diffuse atrophy; affected	♣	Splenium; affected	Diffuse atrophy; affected
Internal capsule	Anterior limb † & posterior limb; affected	♣	Anterior limb & posterior limb; affected	Anterior limb; affected
Basal ganglia	Globus pallidus, caudate nucleus, putamen; affected/unaffected	Globus pallidus, caudate nucleus, putamen; affected /unaffected	Caudate nucleus, putamen; affected	Caudate nucleus, putamen; affected
Thalamus	Unaffected/affected †	Affected	Affected	Unaffected
Substantia nigra	Unaffected	Affected	Affected	Unaffected
Dentate nucleus	Affected	Affected †	Unaffected	Unaffected
Cerebral peduncle	Unaffected	Affected	Affected	Unaffected
Cerebellar peduncles	Affected	Unaffected/affected †	Affected	Affected
Mid brain	Tegmentum, pyramidal tract, medial lemniscus; affected	Periaqueductal region, tegmentum; affected	Periaqueductal region, tegmentum; affected	Unaffected
Pons	Pyramidal tract, medial lemniscus, superior cerebellar peduncle, intraparenchymal part V cranial nerve, mesencephalic trigeminal tract; affected	Affected ♣	Pyramidal tract, medial lemniscus, superior cerebellar peduncle; affected	Superior cerebellar peduncle; affected
Medulla oblongata	Pyramidal tract, medial lemniscus, inferior cerebellar peduncle, anterior spinocerebellar tracts; affected	Affected ♣	Pyramidal tract, medial lemniscus, spinocerebellar tracts; affected	Inferior cerebellar peduncle, high signals; affected
Spinal cord	Dorsal columns & lateral corticospinal tracts of the spinal cord; affected	Unaffected/affected †♣	Dorsal columns & lateral corticospinal tracts; affected	Central part of the spinal cord, affected
Cerebellar white matter	Affected	Affected	Unaffected	Unaffected
Cerebral atrophy	Unaffected	Affected	Unaffected	Affected
Cerebellar atrophy	Affected	Affected	Unaffected	Unaffected
Gene	*DARS2, ISCA2*	>75 genes	*NDUFV1*	*NDUFV1*
References	Toldo et al. (2018) [9], Steenweg et al. (2012) [13]	Rahman et al. (1996) [15], Bonfante et al. (2016) [17]	This paper	This paper

† less common; ♣ not specified.
